# Facial palsy in children: long-term outcome assessed face-to-face and follow-up revealing high recurrence rate

**DOI:** 10.1007/s00405-020-06476-9

**Published:** 2020-12-15

**Authors:** Mervi Kanerva, Hanna Liikanen, Anne Pitkäranta

**Affiliations:** 1 Department of Otorhinolaryngology-Head and Neck Surgery, Helsinki University Hospital and University of Helsinki, Helsinki, Finland; 2 Department of Orthopedics and Traumatology, Helsinki University Hospital and University of Helsinki, Helsinki, Finland

**Keywords:** Facial paralysis, Sunnybrook, House–Brackmann, Facial Nerve Grading System 2.0, Synkinesis, Child

## Abstract

**Purpose:**

To evaluate the long-term (minimum of 2 years from the palsy onset) outcome of pediatric facial palsy by patient questionnaire and face-to-face assessment by the Sunnybrook facial grading system, House–Brackmann grading system, and Facial Nerve Grading System 2.0. To compare the outcome results of self-assessment with the face-to-face assessment. To assess the applicability of the grading scales. To assess the palsy recurrence rate (minimum of a 10-year follow-up).

**Methods:**

46 consecutive pediatric facial palsy patients: 38 (83%) answered the questionnaire and 25 (54%) attended a follow-up visit. Chart review of 43 (93%) after a minimum of 10 years for the facial palsy recurrence rate assessment.

**Results:**

Of the 25 patients assessed face-to-face, 68% had totally recovered but 35% of them additionally stated subjective sequelae in a self-assessment questionnaire. Good recovery was experienced by 80% of the patients. In a 10-year follow-up, 14% had experienced palsy recurrence, only one with a known cause. Sunnybrook was easy and logical to use, whereas House–Brackmann and the Facial Nerve Grading System 2.0 were incoherent.

**Conclusions:**

Facial palsy in children does not heal as well as traditionally claimed if meticulously assessed face-to-face. Patients widely suffer from subjective sequelae affecting their quality of life. Palsy recurrence was high, much higher than previously reported even considering the whole lifetime. Of these three grading systems, Sunnybrook was the most applicable.

## Introduction

If peripheral facial palsy (FP) does not recover fully, cosmetic drawback for the patient is obvious, but the effect on vision, eating, drinking, psychological issues, self-esteem, and quality of life are easily overlooked, although well established [1]. This applies also to situations where voluntary muscle function has recovered quite well but the patient is nonetheless affected by sequelae [2, 3]. Synkineses (voluntary movements accompanied by involuntary movements, e.g., eye closure with mouth movements), contractures, and hemifacial spasms all are post-palsy sequelae.

It has been a common claim that children recover well from FP. This claim has been based on retrospective studies mostly with short follow-up periods [4–11]. Prospective studies on solely pediatric FP patients are sparse.

A reliable grading method is required to be able to assess and communicate FP outcome, and this is currently lacking. Objective methods are being developed but are not yet in wider clinical use. The most used subjective method is the House–Brackmann grading system (HB) (Table 1) [12], although its suitability for present-day demands has been questioned [13–15]. A revised version, the Facial Nerve Grading System 2.0 (FNGS) (Table 2) [16] was developed but has not gained popularity. On the other hand, the use of the Sunnybrook facial grading system (SB) (Table 3) [17, 18] is growing among investigators [14, 19].

**Table 1:** House–Brackmann grading system [12]

Grade	Description	Characteristics
I	Normal	Normal facial function in all areas
II	Mild dysfunction	*Gross*: Slight weakness noticeable on close inspection; may have very slight synkinesis *At rest*: Normal symmetry and tone *Motion*: Forehead: moderate to good function Eye: complete closure with minimal effort Mouth: slight asymmetry
III	Moderate dysfunction	*Gross*: Obvious but not disfiguring difference between two sides; noticeable but not severe synkinesis, contracture, and/or hemifacial spasm *At rest*: Normal symmetry and tone *Motion*: Forehead: slight to moderate movement Eye: complete closure with effort Mouth: slightly weak with maximum effort
IV	Moderately severe dysfunction	*Gross*: Obvious weakness and/or disfiguring asymmetry *At rest*: Normal symmetry and tone *Motion*: Forehead: none Eye: incomplete closure Mouth: asymmetric with maximum effort
V	Severe dysfunction	*Gross*: Only barely perceptible motion *At rest*: Asymmetry *Motion*: Forehead: none Eye: incomplete closure Mouth: slight movement
VI	Total paralysis	No movement

**Table 2:** Facial Nerve Grading System 2.0 [16]

Region					
Score	Brow	Eye		Nasolabial fold	Oral
1	Normal	Normal		Normal	Normal
2	Slight weakness > 75% of normal	Slight weakness > 75% of normal Complete closure with mild effort		Slight weakness > 75% of normal	Slight weakness > 75% of normal
3	Obvious weakness > 50% of normal Resting symmetry	Obvious weakness > 50% of normal Complete closure with maximal effort		Obvious weakness > 50% of normal Resting symmetry	Obvious weakness > 50% of normal Resting symmetry
4	Asymmetry at rest < 50% of normal	Asymmetry at rest < 50% of normal Cannot close completely		Asymmetry at rest < 50% of normal	Asymmetry at rest < 50% of normal
5	Trace movement	Trace movement		Trace movement	Trace movement
6	No movement	No movement		No movement	No movement
Secondary movement (global assessment)					
Score			Degree of movement		
0 1 2 3			None Slight synkinesis; minimal contracture Obvious synkinesis; mild to moderate contracture Disfiguring synkinesis; severe contracture		
Reporting: sum scores for each region and secondary movement					
Grade I II III IV V VI			Total score 4 5–9 10–14 15–19 20–23 24

**Table 3:** Sunnybrook facial grading system [17, 18]

Sunnybrook facial grading system										
Resting symmetry Compared with normal side	Symmetry of voluntary movement Degree of muscle excursion compared with normal side						Synkinesis Rate the degree of involuntary muscle contraction associated with each expression			
Eye (choose one only) Normal 0 Narrow 1 Eyelid surgery 1 Wide 1 Cheek (nasolabial fold) Normal 0 Absent 2 Less pronounced 1 More pronounced 1 Mouth Normal 0 Corner drooped 1 Corner pulled up/out 1	Standard expressions	Unable to initiate movement	Initiates slight movement	Initiates movement with mild excursion	Movement almost complete	Movement complete	None^a^	Mild^b^	Moderate^c^	Severe^d^
	Brow lift	1	2	3	4	5	0	1	2	3
	Gentle eye closure	1	2	3	4	5	0	1	2	3
	Open mouth smile	1	2	3	4	5	0	1	2	3
	Snarl	1	2	3	4	5	0	1	2	3
	Lip pucker	1	2	3	4	5	0	1	2	3
Total		Gross asymmetry	Severe asymmetry	Moderate asymmetry	Mild asymmetry	Normal symmetry				
Resting symmetry score: Total × 5						Total				
	Voluntary movement score: Total × 4						Synkinesis score: Total			
Voluntary movement score − resting symmetry score − synkinesis score = composite score										

^a^No synkinesis or mass movement

^b^Slight synkinesis of one or more muscles

^c^Obvious synkinesis of one or more muscles

^d^Disfiguring synkinesis/gross mass movement of several muscles

The aim of this study was to assess the long-term (minimum of 2 years) outcome of pediatric FP using a patient/caregiver self-assessment FP outcome questionnaire and by face-to-face grading using HB, FNGS, and SB concomitantly. The second aim was to compare the applicability of the grading scales. Third, we wanted to know whether patient questionnaire self-assessed FP outcome results varied from gradings performed by the investigator. Fourth, after a minimum of 10 years from the FP onset, we assessed from the patient charts whether any new FP recurrences had occurred.

## Material and methods

A prospective study of FP in children, with 46 children (≤ 16 years), was conducted between May 2007 and August 2009 [20]. After a minimum of 2 years from the palsy onset, the study patients were requested to participate in a follow-up study. The recruitment letter contained an invitation to come for a one-time face-to-face FP grading session and a questionnaire for self/caregiver-assessment of the long-term FP outcome, recovery, and possible sequelae results; it also included the Synkinesis Assessment Questionnaire (SAQ) [21] (Tables 4, 5). Should the patients not wish to take part in the clinical examinations, they were able to take part in the study by returning the completed questionnaire.

**Table 4:** Synkinesis Assessment Questionnaire [21]

Please answer the following questions regarding facial function, on a scale from 1 to 5, according to the following scale:
1 = seldom or not at all
2 = occasionally, or very mildly
3 = sometimes, or mildly
4 = most of the time, or moderately
5 = all the time, or severely
A. When I smile, my eye closes
B. When I speak, my eye closes
C. When I whistle or pucker my lips, my eye closes
D. When I smile, my neck tightens
E. When I close my eyes, my face gets tight
F. When I close my eyes, the corner of my mouth moves
G. When I close my eyes, my neck tightens
H. When I eat, my eye waters
I. When I move my face, my chin develops a dimpled area

**Table 5:** Study questionnaire (in addition to Synkinesis Assessment Questionnaire, see Table 4)

1. You had/your child had had facial palsy at least once at the time of our previous study. Have you/your child experienced facial palsy recurrence after that time? If so, when.
2. Are there any relatives who also have had facial palsy? If so, mark the kinship, no personal data of that person.
3. Do you/does your child have any of the following sequelae symptoms?
A. Post-palsy functional defects affecting speaking
B. Post-palsy functional defects affecting eating or drinking
C. Clicking or other sounds in the ear produced by mouth movements
D. Excessive tearing
E. Decreased tearing/dry eyes
F. Dry mouth
G. Facial area aches
H. Increased sensitivity to normal sounds
I. Something else, what? (Sequelae affecting outlooks, social situations, etc.)
4. Have you/your child fully recovered from facial palsy?
5. Do you/your child have any other diseases, if so, what?
6. Do you/your child use any regular medications, if so, what?

In January 2020, after a minimum of 10 years from the palsy onset, the patient records for all the 46 patients taking part in the initial prospective study [20] were viewed to see whether any new palsy recurrences had appeared. If the patient did not take part in this current long-term outcome follow-up study, the outcome mentioned in the patient charts was recorded.

At the one-time visit, the corresponding author assessed the FP long-term outcome by the HB, FNGS, and SB grading methods.

For the prospective part (questionnaire and follow-up visit), the Helsinki University Hospital Ethics Committee approved the study, institutional research approval was granted, and all patients gave their written informed consent. For the retrospective chart study, the Department of Otorhinolaryngology-Head and Neck Surgery, Helsinki University Hospital, institutional research approval was requested and granted.

We used descriptive statistics to summarize the frequencies, proportions, means or medians, and ranges.

## Results

### Patient characteristics

Of the 46 patients, 38 (83%) answered the questionnaire, and 25 (54%) both answered the questionnaire and came to the follow-up visit.

At the time of FP onset, the median age of the 38 participants was 10.75 years (range 0.04–16.2 years). Of them, 21 (55%) were girls and 17 (45%) were boys. FP was on the right side for 21 (55%) cases, the left side for 15 (39%), and was bilateral for 2 (5%).

The median follow-up time since FP onset to the evaluation of FP outcome was 3.5 years (range 2.3–4.6 years).

At the time of the chart review for the FP recurrence evaluation, 10.3–12.6 years had passed from the FP onset.

### Facial palsy outcome

#### Questionnaire

In the SAQ (Tables 6, 7), 14/37 (38%) reported synkineses (1 child/caregiver had left the synkinesis part of the questionnaire blank). Other sequelae (Tables 5, 7) were reported by 15/38 (39%) patients as follows: 3/38 (8%) sequelae affecting speaking, 3/38 (8%) sequelae affecting eating or drinking, 4/38 (11%) oro-aural synkinesis (mouth movements accompanied by clicking sounds in the ear), 4/38 (11%) excessive tearing, 4/38 (11%) dry eyes, 1/38 (3%) dry mouth, 1/38 (3%) facial ache, and 1/38 (3%) hyperacusis (increased sensitivity to normal sounds). Additionally, 9/38 (24%) had sequelae that hindered outlook or social situations.

**Table 6:** Synkinesis Assessment Questionnaire results from 37/38 patients (one unanswered)

	Severity 1† Number of patients (%)	Severity 2† Number of patients (%)	Severity 3† Number of patients (%)	Severity 4† Number of patients (%)	Severity 5† Number of patients (%)
A. When I smile, my eye closes	30 (81)	0	3 (8)	1 (3)	3 (8)
B. When I speak, my eye closes	33 (89)	0	3 (8)	0	1 (3)
C. When I whistle or pucker my lips, my eye closes	29 (78)	3 (8)	1 (3)	1 (3)	3 (8)
D. When I smile, my neck tightens	33 (89)	3 (8)	1 (3)	0	0
E. When I close my eyes, my face gets tight	33 (89)	2 (5)	1 (3)	1 (3)	0
F. When I close my eyes, the corner of my mouth moves	33 (89)	1 (3)	0	1 (3)	2 (5)
G. When I close my eyes, my neck tightens	37 (100)	0	0	0	0
H. When I eat, my eye waters	36 (97)	1 (3)	0	0	0
I. When I move my face, my chin develops a dimpled area	32 (86)	2 (5)	0	1 (3)	2 (5)

^†^1 = seldom or not at all; 2 = occasionally, or very mildly; 3 = sometimes, or mildly; 4 = most of the time, or moderately; 5 = all the time, or severely

**Table 7:** Facial palsy assessment results: face-to-face and self-assessment

Patient number	SB^a^	FNGS^b^	HB^c^	Facial palsy occurrences	Self-assessment: full recovery	Self-assessment: synkinesis (see Tables 4, 6)	Self-assessment sequelae remarks (see Table 5, question 3 A–I)
1	98 (100-0-2)	II/5	I, II^e^	1	Yes	D3,E2,I2	I: facial asymmetry
2	94 (96-0-2)	II/6	II	1	No	A5,B5,C5	–
3	91 (96-5-0)	II/5	II	1	No	F2	I: facial asymmetry
4	78 (84-5-1)	II/7	II, III, IV^e^	3	No	Unanswered	–
5	64 (84-10-10)	II/8	II, V^e^	Congenital	No	A3,B3,C4,I2	A, I: outlook
6	60 (72-5-7)	III/12	II, V^e^	1	No	C2,D2,E4,F4,I4	A, B, C, E, F, H, I: weak mouth corner
7	48 (60-5-7)	III/13^d^	III	2	No	A5,C5,F5,I5	B, E
8	37 (44-0-7)	IV/19^d^	IV	2	No	A5,C5,F5,I5	B, E, I: sequelae irritates, gets bullied
9	100	I/4	I	1	No	A4,B3,D2,E3	A, E, I: twitching of the head
10	100	I/4	I	1	No	D2	I: facial tightness, functional asymmetry
11	100	I/4	I	1	No	C2	I: eye function decreased
12	100	I/4	I	1	Yes	E2,H2	D
13	100	I/4	I	1	Yes	A3,C2	C, D, G
14	100	I/4	I	2	Yes	A-I 1	C, D
15	100	I/4	I	1	Yes	A-I 1	–
16	100	I/4	I	1	Yes	A-I 1	–
17	100	I/4	I	1	Yes	A-I 1	–
18	100	I/4	I	1	Yes	A-I 1	–
19	100	I/4	I	1	Yes	A-I 1	–
20	100	I/4	I	1	Yes	A-I 1	–
21	100	I/4	I	1	Yes	A-I 1	–
22	100	I/4	I	1	Yes	A-I 1	–
23	100	I/4	I	1	Yes	A-I 1	–
24	100	I/4	I	1	Yes	A-I 1	–
25	100	I/4	I	1	Yes	A-I 1	–
26	^f^	^f^	^f^	1	No	A3,B3,C3	I: facial asymmetry when smiling
27	^f^	^f^	^f^	1	No	A3,E2	D
28	^f^	^f^	^f^	1	Yes	A-I 1	C
29	^f^	^f^	^f^	1	Yes	A-I 1	–
30	^f^	^f^	^f^	1	Yes	A-I 1	–
31	^f^	^f^	^f^	1	Yes	A-I 1	–
32	^f^	^f^	^f^	1	Yes	A-I 1	–
33	^f^	^f^	^f^	1	Yes	A-I 1	–
34	^f^	^f^	^f^	1	Yes	A-I 1	–
35	^f^	^f^	^f^	1	Yes	A-I 1	–
36	^f^	^f^	^f^	1	Yes	A-I 1	–
37	^f^	^f^	^f^	1	Yes	A-I 1	–
38	^f^	^f^	^f^	1	Yes	A-I 1	–

^a^Sunnybrook facial grading system: total score 0–100 (voluntary movement − resting symmetry − synkinesis)

^b^Facial Nerve Grading System 2.0: grade I–VI/total score 4–24

^c^House–Brackmann grading system (HB): grade I–VI

^d^Discrepancy between voluntary muscle function grade and resting symmetry grade; latter ignored

^e^Impossible to assign the patient to only one HB grade; discrepancy between findings

^f^Questionnaire only

#### Face-to-face assessment

##### *Full recovery, subjective sequelae*

The FP outcome assessment was performed meticulously, also taking into account the slight synkineses or functional defects. Of the 25 patients assessed, 17 (68%) were found by the assessor to be fully recovered (Table 7). Nonetheless, three of these patients/their caregivers had stated in the questionnaire that they did not feel fully recovered. They stated functional defects, synkineses in the SAQ (Tables 6, 7), and other sequelae (Table 7), although at the time of the assessment, these symptoms could not be detected by the assessor. An additional three patients found by the assessor to be fully recovered and who had also checked the full recovery box in their questionnaire, stated some synkineses in the SAQ (two patients, Tables 6, 7) and other sequelae (all three patients, Table 7). Hence, out of those assessed by the investigator to be fully recovered, 5/17 (29%) reported synkineses in the SAQ and 6/17 (35%) other additional sequelae in the self-assessment questionnaire.

##### *Non-full recovery*

From the face-to-face assessment, 8/25 (32%) of the patients had not fully recovered (Table 7): 7/25 (28%) had voluntary muscle function defects, 7/25 (28%) had synkineses, and 5/25 (20%) had asymmetry at rest. Three patients had an SB total score ≥ 90; thus they were close to full recovery but not quite. Including them, 20/25 (80%) patients had recovered very well, although not totally. The remaining 5/25 (20%) patients had an SB total score ≤ 80 (Table 7), here listed with the associative etiologic factor and the SB total score: neurofibromatosis, 37; varicella-zoster virus, 48; neuroborreliosis, 60; congenital, 64; and influenza A, 78.

##### *Applicability of the grading scales*

It was impossible to place the FP outcome in only one HB grade for 4/8 (50%) of the assessed patients who did not recover fully (Table 7). The voluntary movement outcome result was in disagreement with asymmetry at rest and/or synkinesis, and each finding would have placed the patient in a different HB grade. Accordingly, the use of FNGS could not be carried out without ignoring resting symmetry findings, which were in disagreement with the voluntary movement results in 2/8 (25%) patients (Table 7). In other cases, FNGS and SB results were in concordance. FNGS proved to be better than HB, but SB was the most applicable of the three grading scales used.

##### *Medications*

All patients with borreliosis received antibiotic treatment [20]. Four teenage patients with Bell’s palsy received prednisolone; two of them recovered fully, two did not [20]. One patient with herpes association and total recovery received virus medication [20].

##### *Age of patients: full recovery versus non-full recovery*

The median age (at the time of the FP onset) for the patients that had fully recovered according to the follow-up visit face-to-face assessment was 13.8 years (range 0.8–15.6 years) and for those non-fully recovered 6.9 years (range 0.04–15.1 years). Since the latter included one congenital FP, full recovery of that child was impossible: excluding her, the median age for those non-fully recovered was 7.5 years (range 1.5–15.1 years).

#### Patient charts

Of those 46 patients taking part in the initial etiology study [20], 8 did not take part in this follow-up study. From their patient charts it could be registered that five of them had fully recovered within a month and three had recovered to HB II, two within a month and one within 2 months from the FP onset (two of them were not scheduled any further follow-up visits and one was lost for follow-ups). We do know synkineses can develop in months to come even in patients whose voluntary muscle function has fully recovered [22]. Thus, we cannot definitely know whether even those five who rapidly fully recovered stayed fully recovered in the long run.

### Etiology and outcome

From face-to-face assessments, we considered “fully recovered” those with an SB score of 100 and from the questionnaire-only patients those who had checked the box “fully recovered” (Table 7).

Neuroborreliosis was the main etiologic factor in 12/38 (32%) of the patients studied. They all received antibiotic treatment [20]. Of them, six came to the face-to-face assessment and 4/6 (67%) were fully recovered. Six answered only the questionnaire and 5/6 (83%) assessed themselves to be fully recovered. When face-to-face assessment and self-assessment results are combined, of the fully recovered patients 9/28 (32%) were borreliosis patients and of the non-fully recovered patients 3/10 (30%).

Association with varicella-zoster virus was present in 4/38 (11%) of the cases [20]. Of them, 3/4 (75%) recovered fully. Association with human herpesvirus-6 and herpes simplex virus-1 was in 4/38 (11%) [20], of whom 3/4 (75%) recovered fully. Only one of them had received virus medication. Influenza A was detected at the same time as FP in 3/38 (8%) of the patients [20] with 2/3 (67%) recovering fully. The one who did not recover fully had two additional recurrences of FP on the contralateral side without an associative etiologic finding those times. Acute otitis media was detected in 2/38 (5%) patients, both receiving antibiotic treatment [20]. One of them recovered fully, the other one was left with mild synkineses and voluntary movement deficiency. One patient had an association with mycoplasma pneumonia as an etiologic factor [20] and was left with slight synkineses. One patient had congenital FP and 1 patient had neurofibromatosis type II [20] and recurring FP without total recovery. Etiology was unknown in 10/38 (26%) of the cases [20] and 9/10 (90%) recovered fully.

### Facial palsy recurrence

At the time of the questionnaire and assessment visit, 2.3–4.6 years from the initial FP onset, 4 of the 38 (11%) patients had had FP recurrence (Table 7). Two had experienced it on the same side of the face as the initial palsy, two on the contralateral side. The etiology for the recurrence was known for only one patient (neurofibromatosis). As mentioned previously, one patient had already had two recurrences (contralateral side to the initial palsy) without a known cause. In January 2020, patient charts for 43 of the initial 46 patients could be retrieved, 3 had moved out of the district. An additional 2 patients, who were among the 38 patients taking part in this follow-up study, had had a recurrence of FP after the follow-up questionnaire/visit. Thus, at the 10.3–12.6-year follow-up, at least 6/43 (14%) of these children, teenagers, or now young adults had experienced an FP recurrence, only one with a known cause. The number could be even higher if someone had suffered recurrent FP without making contact with the local health-care system.

## Discussion

### Applicability of the grading scales

HB failed to be executable with half of the patients that had any sequelae findings as was FNGS in a quarter of the cases. We have previously shown in our own studies [13, 23, 24] and many colleagues in theirs [14, 15, 25] that often the HB result is forced to be a compromise of voluntary muscle functions (forehead, eye, and mouth) since the recovery levels of different areas of the face can differ greatly. Additionally, resting symmetry and/or synkinesis findings that are in disagreement with the grade best representing the voluntary muscle function status must be ignored to be able to place the patient in only one HB grade. Thus, the interpretation of the scale is only known to the user. FNGS cannot be promoted either because of its inability to take certain resting symmetry findings into consideration since voluntary muscle function grade is linked to resting symmetry grade. From the three grading scales used, SB was the most applicable. Recent studies show SB gaining popularity especially when synkineses are assessed [14, 19].

Many of the studies on grading FP outcomes have used video recordings but investigators have also shown that grading face-to-face with the patient compared with grading from a video affects the results obtained especially concerning the grading of synkinesis [26]. There certainly is a definite need for an objective grading method for FP outcome.

### Facial palsy outcome

Previous studies have suggested that younger children recover better from FP than older children do [5, 27–29]. In our face-to-face study, this was not true: the median age was much higher for the fully recovered children. Our non-fully recovered group was small (eight children) though, with each child’s age having a significant effect, so the end result could be just a coincidence. However, it might not be, since studies contradicting the younger-the-better outcome also exist [10, 30].

We know from previous studies that assessments performed by the professionals differ from a patient’s own assessments—both better and worse [31–33]. Sometimes the patients/caregivers have not noticed slight synkineses seen by the investigator. Also, it is easy to understand that the face can appear and function normally but still ache or feel stiff or weak, also addressing the fact that none of the grading scales used measures a patient’s own sensations. The discrepancy between the questionnaire and face-to-face assessment of FP outcome was clearly shown in this study, which highlights the need to take this into consideration when making conclusions about FP outcome from different types of studies [11, 28].

We question the previous assumptions that almost all children recover well from FP [4–11, 28] based on our current study and previous follow-up studies where the outcome has actually been assessed after at least 1 year from FP onset and face-to-face with the patient, not based on retrospective data from patient charts. Biebl et al. [34] recruited 56 (40%) of 141 pediatric FP patients for a follow-up visit with a result of 44/56 (79%) totally recovered to HB I grade. Nonetheless, they mention that slight weakness and facial asymmetry were noticeable in up to 50% of the patients. The patients had been assigned to HB grade I based on the voluntary muscle function recovery state and ignoring the sequelae findings, which disagreed. In their study, only 40% of the patients arrived for the follow-up visit. They ponder whether that was because the patients had recovered well or because they had remaining symptoms and did not want to be reminded of them. Similarly, in a study by Bagger-Sjöbäck et al. [33], of patients who were considered totally cured at 6 months, 3–5 years later 13% reported subjective symptoms and about half demonstrated signs of sequelae when assessed. In a previous study from our hospital, 69–77% of the pediatric FP patients recovered fully based on face-to-face assessment (subjective symptoms were not asked for nor was a questionnaire used) [35].

Similarly, based on assessment, Skogman et al. [36] found 78% of the patients to be fully recovered, no questionnaire for subjective symptoms was used. In our present study, with meticulous reporting of also slight sequelae, 68% of the patients had recovered totally, but 35% of them reported subjective sequelae invisible to the assessor. Thus, there are now at least three separate studies on pediatric long-term FP outcome showing that 30–50% of patients whose voluntary muscle functions have recovered well experience at least subjective sequelae, and when meticulously assessing the FP outcome, they show mild to moderate sequelae also noticeable by the assessor. We can ask whether those findings are significant and the study by Biebl et al. [34] answers that they are—the social impact for the individual patient was high.

We conclude that in retrospective studies the full recovery and very good outcome results have often been joined, since they are from patient charts and not that specific. Results of 90–100% recovery are predominant. Instead, when the long-term outcome is assessed face-to-face the full recovery result drops to 68–78% and even good recovery (very mild sequelae permitted) to 80–85%, still leaving additional subjective sequelae unreported. These recovery rates resemble those of adults achieved by the same grading method as used in this study (SB) [22].

### Etiology and outcome

When considering the outcome of FP based on etiology in this study, the patient numbers in each etiology category were too small to draw reliable conclusions. Previously it has been shown that the recovery of borreliosis FP patients does not differ from other etiologies [34–36] and that was the result also in this study.

### Facial palsy recurrence

The very surprising result of our study was the recurrence rate: 14% in a 10-year follow-up and 11% within less than 5 years. That is a lot compared with the 6–13% reported previously for the whole lifetime in adults [28, 29]. It is hard to get a clear picture from the previous pediatric FP studies of how the information on recurrence rates has been gained, many of them not mentioning the follow-up time period. The following numbers could be found from the literature for the recurrence: 5.6% [27], 6% [37], 6.8% [38], 6.2% [8], 9% [4]. Two studies from the same investigators have reported high recurrence numbers (25%, 10%), but the numbers seem to reflect more the type of patients that have been referred to them [6, 39]. In our study, the 46 patients that took part in the initial prospective study [20] were consecutive pediatric FP patients from the whole district and the recurrence rate of their FP could be followed over 10 years. Considering this, our 14% recurrence rate was very high and surprising.

## Strengths and limitations

The strength of this study was the consecutive nature of the patients from a previous pediatric facial palsy prospective study. We treated all pediatric facial palsy patients from the area in our tertiary referral center at the time of the study. Hence, the etiologies and facial palsy outcome should have reflected the objective reality.

The prospective study basis also brought limitations to the study. Facial palsy in children is far less frequent than in adults and our study patient number was low. The study was underpowered to find a statistically significant difference compared with studies reporting 90% or better recovery. For that, we would have needed at least 82 patients (calculations not shown). However, from a treating physician’s perspective, our finding of a 70% recovery rate was quite different in comparison with the 90–100% reported in retrospective studies and, as such, we feel this has clinical significance.

## Conclusions

Not all children recovered well from FP when meticulously assessed face-to-face. Patients also widely suffered from subjective sequelae affecting their quality of life. Even considering the whole lifetime, the observed palsy recurrence rate of 11% after 5 years and 14% after 10 years was surprisingly high, much higher than previously reported. SB was the most recommendable of the grading scales used.
